# Normative Intercorrelations Between EEG Microstate Characteristics

**DOI:** 10.1007/s10548-023-00988-3

**Published:** 2023-07-14

**Authors:** Tobias Kleinert, Kyle Nash, Thomas Koenig, Edmund Wascher

**Affiliations:** 1 Department of Ergonomics, Leibniz Research Centre for Working Environment and Human Factors, Ardeystr. 67, 44139 Dortmund, Germany; 2 Department of Biological Psychology, Clinical Psychology, and Psychotherapy, University of Freiburg, Stefan- Meier Str. 8, 79104 Freiburg, Germany; 3 Department of Psychology, University of Alberta, Edmonton, AB T6G 2E9 Canada; 4 Translational Research Center, University Hospital of Psychiatry, University of Bern, CH-3000 Bern, Switzerland

**Keywords:** EEG microstates, Neural networks, Temporal dynamics, Normative correlations, Microstate C, Global field power (GFP)

## Abstract

EEG microstates are brief, recurring periods of stable brain activity that reflect the activation of large-scale neural networks. The temporal characteristics of these microstates, including their average duration, number of occurrences, and percentage contribution have been shown to serve as biomarkers of mental and neurological disorders. However, little is known about how microstate characteristics of prototypical network types relate to each other. Normative intercorrelations among these parameters are necessary to help researchers better understand the functions and interactions of underlying networks, interpret and relate results, and generate new hypotheses. Here, we present a systematic analysis of intercorrelations between EEG microstate characteristics in a large sample representative of western working populations (*n* = 583). Notably, we find that microstate duration is a general characteristic that varies across microstate types. Further, microstate A and B show mutual reinforcement, indicating a relationship between auditory and visual sensory processing at rest. Microstate C appears to play a special role, as it is associated with longer durations of all other microstate types and increased global field power, suggesting a relationship of these parameters with the anterior default mode network. All findings could be confirmed using independent EEG recordings from a retest-session (*n* = 542).

**Supplementary Information:**

The online version contains supplementary material available at 10.1007/s10548-023-00988-3.

EEG microstates remain stable for short periods of time (~ 40–120ms) before rapidly switching into another network type and can be identified based on resting EEG recordings (for a review, see Michel and Koenig 2018). Four to five prototypical microstate types with distinct topographic appearances (labeled A, B, C, D, and E) typically account for more than 70% of variance in the EEG resting state signal (Koenig et al. 2002; Kleinert et al. 2023; Tarailis et al. 2023). The temporal characteristics of microstates, including their average *duration* in milliseconds, their average number of *occurrences* per second, and their percentage *contribution* to the EEG, have been found to reflect momentary mental states (e.g.Nash et al. 2022b; Bréchet and Michel 2022) and stable trait characteristics (e.g.Schiller et al. 2020; Zanesco et al. 2020; Kleinert and Nash 2022; Kleinert et al. 2022; Nash et al. 2022a). These temporal characteristics may also be used as biomarkers of mental and neurological disorders (e.g., da Cruz et al. 2020; Bochet et al. 2021). For example, patients with schizophrenia show increased contribution of microstate type C (da Cruz et al. 2020). However, it remains unclear how temporal characteristics of prototypical microstate types relate to each other. Normative intercorrelations between microstate characteristics could help researchers understand the functions and interactions of underlying neural networks, interpret and relate their own and previous results, and generate new hypothesis. For example, the contribution of microstate C might show systematic dependencies with temporal characteristics of another prototypical microstate type. These characteristics, and constructs related to the respective microstate type, could then be targeted in follow-up studies on schizophrenia with higher statistical power, and/or using more diverse samples (e.g., with a higher variability in age). In the current study, we use a large sample (*n* = 583) representative of western populations with regard to age, genetics, cognitive abilities, and employment to close this research gap. To investigate the robustness of our findings, all analyses were repeated using an independent EEG dataset from a retest-session after an average interval of 63 days (*n* = 542).

## Results and Discussion

The grand-mean microstate maps of the five microstate types A, B, C, D, and E closely resembled prototypical microstate types from the literature and showed high consistency across both EEG datasets (see Table S1 in the supplementary material). All reported results were robust across both datasets (unless otherwise noted) and all analyses were corrected for multiple testing using the Bonferroni method (see Table S2 in the supplementary material for all results). The code and data used to generate the findings of this study are freely available in the OSF repository (https://osf.io/39w5h/).

### Intercorrelations Between Microstate Durations

**Fig. 1:**
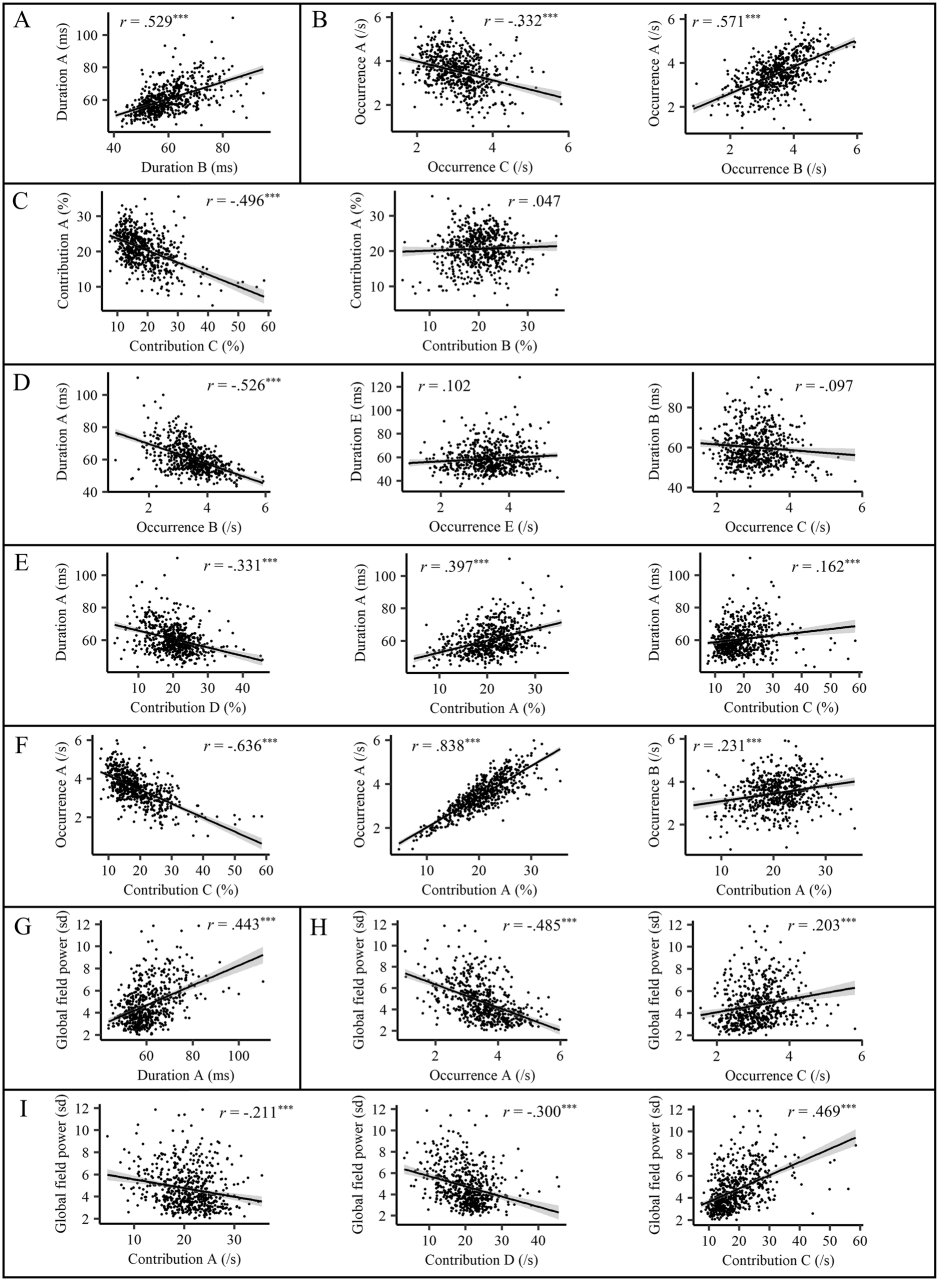
Exemplary intercorrelations of EEG microstate characteristics *n* = 583. ^***^ = p < .001. Shown are scatterplots illustrating exemplary associations between microstate characteristics using EEG data from day one (note that all of these results could be confirmed using data from day two). **A**: Durations of all microstate types showed positive associations. **B**: Occurrences mostly showed negative associations (left plot), but occurrences of **A**, **B**, and **D** showed positive associations (right plot). **C**: Contributions mostly showed negative associations (left plot), but contributions of **A** and **B** were not associated. **D**: Durations were mostly negatively associated with occurrences (left plot), except for much lower negative, non-significant, or positive associations with occurrences of the same microstate type (middle plot) and the occurrence of microstate **C** (right plot). **E**: Similarly, durations were mostly negatively associated with contributions (left plot), except for positive associations with contributions of the same microstate type (middle plot) and microstate **C** contributions (right plot). **F**: Occurrences were mostly negatively associated with contributions (left plot), except for positive associations with contributions of the same microstate type (middle plot), and positive associations of occurrence (**B**) with contribution (**A**) and occurrence (A) with contribution (**B**). **G**: Global field power (GFP) was positively associated with the duration of all microstate types. **H**: GFP was mostly negatively associated with occurrences (left plot), except for positive associations with the occurrence of microstate **C** (right plot). **I**: Global field power was negatively associated with contribution **A** (left plot) and contribution **D** (middle plot), and positively associated with contribution **C** (right plot)

First, we analyzed associations between durations of the five prototypical microstate types (e.g., duration A and duration B). Durations were positively associated with each other across microstate types (average *r* = .390; see Fig. 1A) supporting the notion that individuals generally tend to have longer or shorter microstates (also see Khanna et al. 2014; Kleinert and Nash 2022; Kleinert et al. 2022). Previous studies referred to this phenomenon as “mental processing stability”, which is positively related to self-control and negatively related to risk-taking and aggression (Kleinert and Nash 2022; Kleinert et al. 2022).

### Intercorrelations Between Microstate Occurrences

Second, we analyzed associations between microstate occurrences (e.g., occurrence A and occurrence B). Based on the previous result, we expected positive associations across microstate types, as occurrences are a natural antagonist to durations. Accordingly, we found that durations of the microstates A, B, and D showed positive correlations with each other (average *r* = .517; see Fig. 1B). However, the occurrence of microstate C was negatively related to the occurrence of all other microstate types (average *r* = − .221), indicating that microstate C occurrence has a competing relationship with the occurrence of all other microstate types and a special role within prototypical microstate types. Microstate C is negatively associated with task-related processing (Michel and Koenig 2018) and positively associated with alpha power (Férat et al. 2022) and long-range brain connectivity (Rajkumar et al. 2021), and is assumed to represent the anterior default-mode network (aDMN; Michel and Koenig 2018).

### Intercorrelations Between Microstate Contributions

Third, we analyzed associations between contributions of the five prototypical microstate types (e.g., contribution A and contribution B). We found mainly negative correlations (average *r* = − .279; see Fig. 1C), a sensible result given that a higher contribution of any microstate type leaves less time left in the EEG that could be covered by another type. However, there was no negative correlation of microstate A and B contributions (average *r* = .038), which is in line with the positive associations of microstate A and B durations and occurrences. As microstate A and B have been associated with auditory and visual sensory processing, respectively (Michel and Koenig 2018), these results suggest that people show a general tendency towards more or less sensory processing across auditory and visual systems at rest.

### Correlations Between Microstate Durations and Occurrences

Fourth, we analyzed associations of one microstate’s duration with another microstate’s occurrence (e.g., duration A and occurrence B). Naturally, these correlations were mostly negative (average *r* = − 0.555; see Fig. 1D), as longer durations leave less time for more microstates to occur. An exception were durations and occurrences of the same microstate type, which showed much lower negative, non-significant, or even positive correlations (average *r* = .083). This demonstrates that longer duration of a particular microstate type goes along with more frequent occurrence of the same microstate type, i.e., that people show dominance of certain microstate types across duration and occurrence. Again, another exception was the occurrence of microstate C, which showed small or non-significant correlations with durations of all other microstate types (average *r* = −0.129), emphasizing the special role of this microstate.

### Correlations Between Microstate Durations and Contributions

Fifth, we analyzed associations of one microstate’s duration with another microstate’s contribution (e.g., duration A and contribution B). Naturally, durations were positively correlated with contributions of the same microstate type (as contributions are computed as the product of durations and occurrences; average *r* = .633; see Fig. 1E) and negatively correlated with contributions of other microstate types (as longer durations leave less time to be covered by other microstate types; average *r* = − 0.268). Again, the contribution of microstate C showed an exceptional positive correlation with durations of all other microstate types (average *r* = .200). Thus, increased dominance of the aDMN, as indicated by more occurrences and a higher percentage contribution of microstate C, may drive longer durations of microstates across types (and/or vice versa).

### Correlations Between Microstate Occurrences and Contributions

Sixth, we analyzed associations of one microstate’s occurrence with another microstate’s contribution (e.g., occurrence A and contribution B). Occurrences were mostly negatively correlated with contributions of other microstate types (average *r* = − 0.239; see Fig. 1F), as more frequent occurrences of any microstate type leave less time to be covered by another type. An exception were correlations of occurrences with contributions of the same microstate type, which were positive (average *r* = .827). Again, this is a natural association as contributions are computed as the product of a microstate’s duration and occurrence. Another exception were positive associations of microstate A occurrence with microstate B contribution, and microstate B occurrence with microstate A contribution (average *r* = .189), further highlighting a relationship of mutual reinforcement between resting networks related to auditory and visual sensory processing.

### Correlations Between Microstate Characteristics and Global Field Power

Finally, we analyzed associations of microstate characteristics with *global field power* (GFP), indicating the overall strength of the EEG signal in time-frames covered by microstates. Durations of all microstate types showed positive correlations with GFP (average *r* = .457; see Fig. 1G), which is in line with the notion that microstates show longer durations around GFP maxima in the EEG (e.g., Skrandies 1990). Relatedly, occurrences showed mostly negative correlations with GFP (average *r* = − 0.442; see Fig. 1H). Thus, increased GFP might drive longer durations and fewer occurrences of microstates (and/or vice versa). An exception was the occurrence of microstate C, which was positively correlated with GFP (average *r* = .197). Similarly, the contribution of microstate C was also positively correlated with GFP (average *r* = .478; see Fig. 1I), whereas contributions of all other microstate types showed negative (i.e., A and D; average *r* = − 0.277) or inconsistent correlations across datasets (i.e., B and E). Combined with our previous findings on microstate C and other literature (Férat et al. 2022), this suggest that millisecond activation of the aDMN, general microstate duration, GFP, and alpha power are related neural phenomena.

## Conclusion

In sum, we find that the duration of microstates can be regarded as a general characteristic that varies across microstate types, that microstate A and B show a relationship of mutual reinforcement, and that microstate C is related to longer durations of all other microstate types and GFP. How can these findings support future research? Recalling the example from the introduction, the contribution of microstate C has been related to schizophrenia. Our results show that microstate C dominance is related to longer durations of all other microstate types and GFP. Indeed, schizophrenic patients have shown longer durations of microstate B and D (and A on a marginally significant level; da Cruz et al. 2020). Based on normative intercorrelations, one might interpret that these results are directly related to the primary finding, and not independent. Furthermore, future studies could test the hypotheses that schizophrenia is related to longer microstate duration in more than four microstate types, and to neural phenomena associated with microstate C, such as GFP or alpha power. In sum, we discovered considerable dependencies in EEG microstate characteristics that have been largely neglected. These dependencies contribute to our understanding of the functions and interactions of prototypical microstate networks and have the potential to help interpret and relate results from EEG studies and inspire future research.

### Supplementary Information

Below is the link to the electronic supplementary material.

Supplementary material 1 (DOCX 260.8 kb)

## Data Availability

The data and code of this study are freely available in the OSF repository (https://osf.io/39w5h/)
