# Correction: Normative Intercorrelations between EEG Microstate Characteristics

**DOI:** 10.1007/s10548-023-01012-4

**Published:** 2023-10-20

**Authors:** Tobias Kleinert, Kyle Nash, Thomas Koenig, Edmund Wascher

**Affiliations:** 1 Department of Ergonomics, Leibniz Research Centre for Working Environment and Human Factors, Ardeystr. 67, 44139 Dortmund, Germany; 2 Department of Biological Psychology, Clinical Psychology, and Psychotherapy, University of Freiburg, Stefan- Meier Str. 8, 79104 Freiburg, Germany; 3 Department of Psychology, University of Alberta, T6G 2E9 Edmonton, AB Canada; 4 Translational Research Center, University Hospital of Psychiatry, University of Bern, CH-3000 Bern, Switzerland

**Correction to: Brain Topography**

https://link.springer.com/article/10.1007/s10548-023-00988-3

After the article was published online, the authors noticed inadvertent errors in the “Results and Discussion” section:

1. Under the subheading “Intercorrelations Between Microstate Occurrences”, the average correlation coefficient r = .221 should be negative: r = -.221.

The correct sentence reads as follows:

“However, the occurrence of microstate C was negatively related to the occurrence of all other microstate types (average r = -.221), indicating that microstate C occurrence has a competing relationship with the occurrence of all other microstate types and a special role within prototypical microstate types.”

2. Under the subheading “Intercorrelations Between Microstate Contributions”, the average correlation coefficient r = .279 should be negative: r = -.279.

The correct sentence reads as follows:

“We found mainly negative correlations (average r = -.279; see Fig. 1C), a sensible result given that a higher contribution of any microstate type leaves less time left in the EEG that could be covered by another type.”

The original article has been updated.

