# Supplementary material for: Normative Intercorrelations Between EEG Microstate Characteristics
Source: Brain Topogr. 2023 Jul 14;37(2):265–9. doi: 10.1007/s10548-023-00988-3 (PMC10884083; doi:10.1007/s10548-023-00988-3)
Supplement: Supplementary file 1 — Supplementary material 1 (DOCX 260.8 kb) [file 10548_2023_988_MOESM1_ESM.docx]

**Supplementary MaterialTable S1**

*Grand-mean microstate maps*

|  | **Microstate type** | | | | |
| --- | --- | --- | --- | --- | --- |
| **Dataset** | **A** | **B** | **C** | **D** | **E** |
| **Day one** | 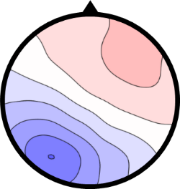 | 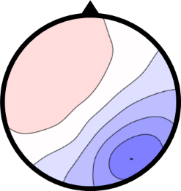 | 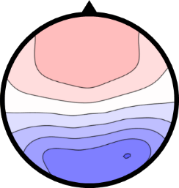 | 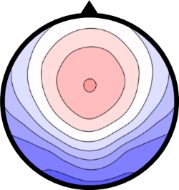 | 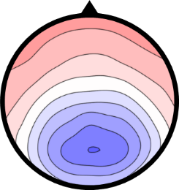 |
| **Day two** | 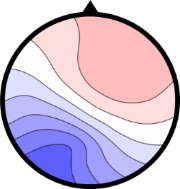 | 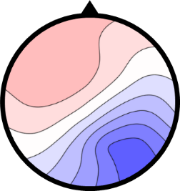 | 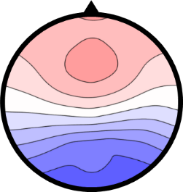 | 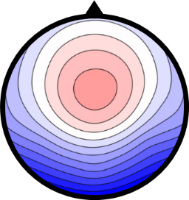 | 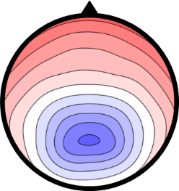 |

*Note.* Day one: *n* = 583, day two: *n* = 542. Grand-mean microstate maps (five clusters) for day one and day two, showing excellent consistency across datasets (spatial correlations of microstate maps of the same type: Microstate A: *r* = .992, Microstate B: *r* = .974, Microstate C: *r* = .996, Microstate D: *r* = .990, Microstate E: *r* = .983.

**Table S2**

*Normative intercorrelations of EEG microstate characteristics*

|  | **Intercorrelations of microstate durations** | | | | | |
| --- | --- | --- | --- | --- | --- | --- |
|  | **Day one** | | | **Day two** | | |
| **Correlation analysis** | r | p | R² | r | p | R² |
| Dur A * Dur B | .529 | <.001 | .280 | .423 | <.001 | .179 |
| Dur A * Dur C | .407 | <.001 | .166 | .409 | <.001 | .168 |
| Dur A * Dur D | .367 | <.001 | .134 | .223 | <.001 | .050 |
| Dur A * Dur E | .456 | <.001 | .208 | .268 | <.001 | .072 |
| Dur B * Dur C | .441 | <.001 | .194 | .514 | <.001 | .264 |
| Dur B * Dur D | .454 | <.001 | .206 | .344 | <.001 | .119 |
| Dur B * Dur E | .412 | <.001 | .170 | .379 | <.001 | .144 |
| Dur C * Dur D | .374 | <.001 | .140 | .370 | <.001 | .137 |
| Dur C * Dur E | .414 | <.001 | .171 | .451 | <.001 | .204 |
| Dur D * Dur E | .281 | <.001 | .079 | .218 | <.001 | .048 |
|  | **Intercorrelations of microstate occurrences** | | | | | |
|  | **Day one** | | | **Day two** | | |
| **Correlation analysis** | r | p | R² | r | p | R² |
| Occ A * Occ B | .571 | <.001 | .326 | .486 | <.001 | .236 |
| Occ A * Occ C | -.332 | <.001 | .110 | -.214 | <.001 | .046 |
| Occ A * Occ D | .461 | <.001 | .213 | .361 | <.001 | .130 |
| Occ A * Occ E | .321 | <.001 | .103 | -.002 | 1.00 | <.001 |
| Occ B * Occ C | -.247 | <.001 | .061 | -.179 | <.001 | .032 |
| Occ B * Occ D | .514 | <.001 | .265 | .348 | <.001 | .121 |
| Occ B * Occ E | .173 | <.001 | .030 | -.060 | 1.00 | .004 |
| Occ C * Occ D | -.142 | .006 | .020 | -.217 | <.001 | .047 |
| Occ C * Occ E | -.204 | <.001 | .042 | -.228 | <.001 | .052 |
| Occ D * Occ E | .122 | .032 | .015 | .064 | 1.00 | .004 |
|  | **Intercorrelations of microstate contributions** | | | | | |
|  | **Day one** | | | **Day two** | | |
| **Correlation analysis** | r | p | R² | r | p | R² |
| Con A * Con B | .047 | 1.00 | .002 | .028 | 1.00 | .001 |
| Con A * Con C | -.496 | <.001 | .246 | -.371 | <.001 | .138 |
| Con A * Con D | -.137 | .009 | .019 | -.159 | .002 | .025 |
| Con A * Con E | -.136 | .010 | .019 | -.376 | <.001 | .141 |
| Con B * Con C | -.399 | <.001 | .159 | -.268 | <.001 | .072 |
| Con B * Con D | -.004 | 1.00 | <.001 | -.140 | .011 | .020 |
| Con B * Con E | -.337 | <.001 | .114 | -.369 | <.001 | .136 |
| Con C * Con D | -.352 | <.001 | .124 | -.351 | <.001 | .123 |
| Con C * Con E | -.151 | .003 | .023 | -.106 | .133 | .011 |
| Con D * Con E | -.435 | <.001 | .189 | -.345 | <.001 | .119 |
|  | **Correlations of microstate durations and occurrences** | | | | | |
|  | **Day one** | | | **Day two** | | |
| **Correlation analysis** | r | p | R² | r | p | R² |
| Dur A * Occ A | -.149 | .008 | .022 | -.001 | 1.00 | <.001 |
| Dur A * Occ B | -.526 | <.001 | .277 | -.401 | <.001 | .161 |
| Dur A * Occ C | -.196 | <.001 | .038 | -.090 | .882 | .008 |
| Dur A * Occ D | -.594 | <.001 | .352 | -.553 | <.001 | .305 |
| Dur A * Occ E | -.435 | <.001 | .189 | -.406 | <.001 | .165 |
| Dur B * Occ A | -.560 | <.001 | .313 | -.540 | <.001 | .291 |
| Dur B * Occ B | -.196 | <.001 | .038 | -.086 | 1.00 | .007 |
| Dur B * Occ C | -.097 | .482 | .009 | -.067 | 1.00 | .004 |
| Dur B * Occ D | -.568 | <.001 | .323 | -.598 | <.001 | .357 |
| Dur B * Occ E | -.563 | <.001 | .317 | -.372 | <.001 | .138 |
| Dur C * Occ A | -.684 | <.001 | .467 | -.641 | <.001 | .411 |
| Dur C * Occ B | -.685 | <.001 | .469 | -.589 | <.001 | .346 |
| Dur C * Occ C | .398 | <.001 | .158 | .404 | <.001 | .163 |
| Dur C * Occ D | -.653 | <.001 | .426 | -.612 | <.001 | .374 |
| Dur C * Occ E | -.447 | <.001 | .200 | -.388 | <.001 | .150 |
| Dur D * Occ A | -.568 | <.001 | .323 | -.504 | <.001 | .254 |
| Dur D * Occ B | -.476 | <.001 | .227 | -.445 | <.001 | .198 |
| Dur D * Occ C | -.149 | .008 | .022 | -.207 | <.001 | .043 |
| Dur D * Occ D | -.051 | 1.00 | .003 | .064 | 1.00 | .004 |
| Dur D * Occ E | -.583 | <.001 | .340 | -.401 | <.001 | .161 |
| Dur E * Occ A | -.550 | <.001 | .302 | -.632 | <.001 | .399 |
| Dur E * Occ B | -.628 | <.001 | .394 | -.629 | <.001 | .395 |
| Dur E * Occ C | -.142 | .015 | .020 | -.081 | 1.00 | .007 |
| Dur E * Occ D | -.694 | <.001 | .481 | -.622 | <.001 | .386 |
| Dur E * Occ E | .102 | .344 | .010 | .297 | <.001 | .088 |
|  | **Correlations of microstate durations and contributions** | | | | | |
|  | **Day one** | | | **Day two** | | |
| **Correlation analysis** | r | p | R² | r | p | R² |
| Dur A * Con A | .397 | <.001 | .158 | .445 | <.001 | .198 |
| Dur A * Con B | -.194 | <.001 | .038 | -.151 | .011 | .023 |
| Dur A * Con C | .162 | .002 | .026 | .195 | <.001 | .038 |
| Dur A * Con D | -.331 | <.001 | .109 | -.342 | <.001 | .117 |
| Dur A * Con E | -.055 | 1.00 | .003 | -.140 | .028 | .019 |
| Dur B * Con A | -.238 | <.001 | .057 | -.294 | <.001 | .086 |
| Dur B * Con B | .407 | <.001 | .166 | .449 | <.001 | .201 |
| Dur B * Con C | .240 | <.001 | .058 | .277 | <.001 | .077 |
| Dur B * Con D | -.253 | <.001 | .064 | -.309 | <.001 | .096 |
| Dur B * Con E | -.169 | .001 | .028 | -.053 | 1.00 | .003 |
| Dur C * Con A | -.415 | <.001 | .172 | -.377 | <.001 | .142 |
| Dur C * Con B | -.375 | <.001 | .141 | -.250 | <.001 | .062 |
| Dur C * Con C | .876 | <.001 | .767 | .853 | <.001 | .727 |
| Dur C * Con D | -.356 | <.001 | .127 | -.298 | <.001 | .089 |
| Dur C * Con E | -.080 | 1.00 | .006 | -.026 | 1.00 | .001 |
| Dur D * Con A | -.336 | <.001 | .113 | -.350 | <.001 | .123 |
| Dur D * Con B | -.181 | <.001 | .033 | -.216 | <.001 | .047 |
| Dur D * Con C | .164 | .002 | .027 | .105 | .372 | .011 |
| Dur D * Con D | .503 | <.001 | .253 | .580 | <.001 | .336 |
| Dur D * Con E | -.272 | <.001 | .074 | -.172 | .001 | .030 |
| Dur E * Con A | -.263 | <.001 | .069 | -.440 | <.001 | .194 |
| Dur E * Con B | -.340 | <.001 | .116 | -.360 | <.001 | .130 |
| Dur E * Con C | .204 | <.001 | .042 | .246 | <.001 | .060 |
| Dur E * Con D | -.445 | <.001 | .198 | -.386 | <.001 | .149 |
| Dur E * Con E | .681 | <.001 | .464 | .755 | <.001 | .570 |
|  | **Correlations of microstate occurrences and contributions** | | | | | |
|  | **Day one** | | | **Day two** | | |
| **Correlation analysis** | r | p | R² | r | p | R² |
| Occ A * Con A | .838 | <.001 | .703 | .887 | <.001 | .786 |
| Occ A * Con B | .169 | .001 | .028 | .120 | .132 | .014 |
| Occ A * Con C | -.636 | <.001 | .404 | -.531 | <.001 | .282 |
| Occ A * Con D | .059 | 1.00 | .004 | .007 | 1.00 | <.001 |
| Occ A * Con E | -.117 | .116 | .014 | -.348 | <.001 | .121 |
| Occ B * Con A | .231 | <.001 | .053 | .235 | <.001 | .055 |
| Occ B * Con B | .803 | <.001 | .645 | .840 | <.001 | .706 |
| Occ B * Con C | -.595 | <.001 | .354 | -.479 | <.001 | .230 |
| Occ B * Con D | .167 | .001 | .028 | .030 | 1.00 | .001 |
| Occ B * Con E | -.264 | <.001 | .070 | -.387 | <.001 | .149 |
| Occ C * Con A | -.406 | <.001 | .165 | -.221 | <.001 | .049 |
| Occ C * Con B | -.271 | <.001 | .074 | -.181 | .001 | .033 |
| Occ C * Con C | .781 | <.001 | .609 | .810 | <.001 | .657 |
| Occ C * Con D | -.198 | <.001 | .039 | -.280 | <.001 | .078 |
| Occ C * Con E | -.209 | <.001 | .044 | -.185 | <.001 | .034 |
| Occ D * Con A | .087 | .911 | .008 | .052 | 1.00 | .003 |
| Occ D * Con B | .123 | .076 | .015 | -.017 | 1.00 | <.001 |
| Occ D * Con C | -.522 | <.001 | .272 | -.511 | <.001 | .261 |
| Occ D * Con D | .827 | <.001 | .684 | .839 | <.001 | .705 |
| Occ D * Con E | -.344 | <.001 | .118 | -.318 | <.001 | .101 |
| Occ E * Con A | .054 | 1.00 | .003 | -.197 | <.001 | .039 |
| Occ E * Con B | -.173 | .001 | .030 | -.254 | <.001 | .064 |
| Occ E * Con C | -.394 | <.001 | .155 | -.360 | <.001 | .129 |
| Occ E * Con D | -.210 | <.001 | .044 | -.165 | .003 | .027 |
| Occ E * Con E | .786 | <.001 | .618 | .837 | <.001 | .701 |
|  | **Correlations of microstate characteristics and global field power** | | | | | |
|  | **Day one** | | | **Day two** | | |
| **Correlation analysis** | r | p | R² | r | p | R² |
| Dur A * GFP | .443 | <.001 | .197 | .352 | <.001 | .124 |
| Dur B * GFP | .531 | <.001 | .282 | .464 | <.001 | .215 |
| Dur C * GFP | .553 | <.001 | .306 | .604 | <.001 | .364 |
| Dur D * GFP | .331 | <.001 | .110 | .279 | <.001 | .078 |
| Dur E * GFP | .411 | <.001 | .169 | .544 | <.001 | .296 |
| Occ A * GFP | -.485 | <.001 | .235 | -.519 | <.001 | .270 |
| Occ B * GFP | -.412 | <.001 | .170 | -.490 | <.001 | .240 |
| Occ C * GFP | .203 | <.001 | .041 | .191 | <.001 | .037 |
| Occ D * GFP | -.544 | <.001 | .295 | -.527 | <.001 | .278 |
| Occ E * GFP | -.395 | <.001 | .156 | -.109 | .166 | .012 |
| Con A * GFP | -.211 | <.001 | .045 | -.308 | <.001 | .095 |
| Con B * GFP | -.073 | 1.00 | .005 | -.199 | <.001 | .040 |
| Con C * GFP | .469 | <.001 | .220 | .487 | <.001 | .238 |
| Con D * GFP | -.300 | <.001 | .090 | -.286 | <.001 | .082 |
| Con E * GFP | -.046 | 1.00 | .002 | .209 | <.001 | .044 |

Day 1: *n* = 583; Day 2: *n* = 542. r = Pearson correlation coefficient, p = p-value, R² = r-squared value. Dur = Duration, Occ = Occurrence, Con = Contribution, GFP = global field power. Shown are intercorrelations between EEG microstate characteristics (corrected for multiple testing in each section using the Bonferroni method).

**Methods**

**Sample**

All data was collected in the Dortmund Vital Study (DVS; for details, see Gajewski et al. 2022). The study was approved by the local ethics committee at the Leibniz Research Centre for Working Environment and Human Factors and conducted with the informed written consent of participants according to the principles expressed in the Declaration of Helsinki. The data and code used to generate the findings of this study are available in the OSF repository (https://osf.io/39w5h/). The study consisted of two experimental sessions (day one and day two; average interval between sessions: 63 days). After excluding participants due to measurement problems or excessive artifacts in the EEG (>50% data loss), there was a sample of *n* = 583 (363 women, 220 men; age: *M* = 43.83 years, *SD* = 14.30) available for day one analyses (initial sample: *n* = 609). Out of these, *n* = 542 (334 women, 208 men; age: *M* = 43.85 years, *SD* = 14.30) were available for day two analyses.

**Procedure**

Participants were recruited using online, print, and live media. First, participants registered for the DVS using a contact form. Second, they were informed about the study via telephone interviews. Professional lab workers ran both experiments (i.e., day one and day two). On day one, resting EEG with closed and open eyes (three minutes each) were recorded in an EEG cabin (eyes closed) using a 64-channel system (Brain Products, Gilching, Germany; online sampling rate: 1000 Hz, reference electrode: FCz, grounding electrode: AFz; montage according to the extended 10-20 system). On day two, the same procedure was applied using a 30-channel system (BioSemi B. V., Amsterdam, Netherlands; online sampling rate: 2048 Hz, grounding and referencing via a common mode sense (CMS) active electrode and a driven right leg (DRL) passive electrode; montage according to the extended 10-20 system). Impedances for both sessions were below 10 kΩ. The duration of resting EEG measures on day two was two minutes. Only eyes-closed periods were used for further analyses. Note that other tasks were completed on both days that are not relevant for the current study. Participants received a monetary compensation of €160 (day one: €100; day two: €60).

**EEG preprocessing**

We used EEGLAB (Delorme and Makeig 2004) to preprocess the EEG data. The following preprocessing steps were applied: 1) Downsampling to 500 Hz (512 Hz on day two). 2) Band-pass filtering from 2 to 20 Hz (typical frequency range in microstate research; e.g., Koenig et al. 2002). 3) Automated removal of large artifacts using spectrum thresholding (EEGLAB function: pop_rejcont; recommended settings; frequency range: 15-30 Hz). 4) Re-referencing to average (day one only, not necessary using the BioSemi system). 5) Exclusion of noisy EEG channels with the PrepPipeline (Bigdely-Shamlo et al. 2015). 6) Additional artifact correction (EEGLAB function: pop_autorej; recommended settings; threshold for detecting large artifacts: 500 µV). 7) Identification of regular artifacts using an ICA (EEGLAB function: pop_runica; recommended settings). 8) Rejection of components reflecting eye-movements or muscle artifacts (probability >70%; EEGLAB function: ICLabel; Pion-Tonachini et al. 2019).

**EEG microstate analysis**

We computed microstate characteristics using the microstate toolbox for EEGLAB (Koenig 2017; version 1.2). In each individual, electric potential field maps from all local global field power (GFP) maxima were extracted. These maps were submitted to an atomize and agglomerate hierarchical clustering procedure (AAHC; Murray et al. 2008) to identify the most dominant five maps in each individual (these five maps were shown to be most reliable across different samples and methods in a previous study using the same datasets; see Kleinert et al. in press). AAHC was used rather than k-means clustering as using AAHC results in somewhat more reliable microstate characteristics (Kleinert et al. in press). Also note that microstate characteristics obtained from AAHC and k-means clustering are highly consistent (Kleinert et al. in press). Then, the dominant maps of all participants were submitted to another cluster analysis to identify a set of grand-mean microstate maps across participants for each day. These five maps closely resembled prototypical microstate maps known from the literature (e.g., Zanesco et al. 2020; Férat et al. 2022; Kleinert et al. in press), and were labeled accordingly (i.e., A, B, C, D, E; see Table S2). Next, initial electric potential field maps of each participant were assigned to one of the five microstate types based on spatial correlations with the labeled grand-mean microstate maps. Time-points between the GFP maxima were assigned to the same microstate type as their nearest neighbor. This backfitting procedure resulted in a continuous sequence of microstate maps for each individual, from which microstate characteristics were extracted: the average *duration* of each microstate type in milliseconds, the average number of *occurrences* of each microstate type per second, the percentage *contribution* of each microstate type to the variance that can be explained by all microstate types combined, and the mean global field power across all time-frames in the EEG covered by microstates (average standard deviation of the electrical potentials of all channels at each timepoint).

**Statistical analyses**

We computed Pearson correlation coefficients to investigate intercorrelations between the five different microstate types’ durations, occurrences, and contributions (see Table S2 in the supplementary material for all results). All analyses were repeated using data from day two to test for the robustness of our findings. To correct for multiple testing, we applied Bonferroni corrections to p-values in each set of analyses. First, we analyzed intercorrelations between microstate durations of all microstate types (10 correlations [Dur = duration)]: Dur A*Dur B, Dur A*Dur C, Dur A*Dur D, Dur A*Dur E, Dur B*Dur C, Dur B*Dur D, Dur B*Dur E, Dur C*Dur D, Dur C*Dur E, Dur D*Dur E). Then, we repeated this procedure using occurrences and contributions of all microstate types. Next, we analyzed correlations between durations and occurrences (25 correlations [Occ = occurrence]: Dur A*Occ A, Dur A*Occ B, Dur A*Occ C, Dur A*Occ D, Dur A*Occ E, Dur B*Occ A, Dur B*Occ B, Dur B*Occ C, Dur B*Occ D, Dur B*Occ E, Dur C*Occ A, Dur C*Occ B, Dur C*Occ C, Dur C*Occ D, Dur C*Occ E, Dur D*Occ A, Dur D* Occ B, Dur D*Occ C, Dur D*Occ D, Dur D*Occ E, Dur E*Occ A, Dur E* Occ B, Dur E*Occ C, Dur E*Occ D, Dur E*Occ E). This procedure was repeated to analyze correlations between durations and contributions, and occurrences and contributions. Finally, we analyzed correlations between global field power (GFP) and durations, occurrences, and contributions (15 correlations [Con = contribution]: GFP*Dur A, GFP*Dur B, GFP*Dur C, GFP*Dur D, GFP*Dur E, GFP*Occ A, GFP*Occ B, GFP*Occ C, GFP*Occ D, GFP*Occ E, GFP*Con A, GFP*Con B, GFP* Con C, GFP*Con D, GFP*Con E). To report average correlations in the text as a quick reference, we z-transformed correlations, averaged the values, and back-transformed the result (as correlation coefficients are not normally distributed).**References (supplementary material)**

Bigdely-Shamlo N, Mullen T, Kothe C, et al (2015) The PREP pipeline: standardized preprocessing for large-scale EEG analysis. Front Neuroinformatics 9:16

Delorme A, Makeig S (2004) EEGLAB: an open source toolbox for analysis of single-trial EEG dynamics including independent component analysis. J Neurosci Methods 134:9–21

Férat V, Seeber M, Michel CM, Ros T (2022) Beyond broadband: Towards a spectral decomposition of electroencephalography microstates. Hum Brain Mapp

Gajewski PD, Getzmann S, Bröde P, et al (2022) Impact of Biological and Lifestyle Factors on Cognitive Aging and Work Ability in the Dortmund Vital Study: Protocol of an Interdisciplinary, Cross-sectional, and Longitudinal Study. JMIR Res Protoc 11:e32352

Kleinert T, Koenig T, Nash K, Wascher E (in press) On the reliability of the EEG microstate approach

Koenig T (2017) EEGLAB microstate plugin. In: Microstates EEGLAB. https://www.thomaskoenig.ch/index.php/software/microstates-in-eeglab/getting-started

Koenig T, Prichep L, Lehmann D, et al (2002) Millisecond by millisecond, year by year: normative EEG microstates and developmental stages. Neuroimage 16:41–48

Murray MM, Brunet D, Michel CM (2008) Topographic ERP analyses: a step-by-step tutorial review. Brain Topogr 20:249–264

Pion-Tonachini L, Kreutz-Delgado K, Makeig S (2019) ICLabel: An automated electroencephalographic independent component classifier, dataset, and website. NeuroImage 198:181–197

Zanesco AP, King BG, Skwara AC, Saron CD (2020) Within and between-person correlates of the temporal dynamics of resting EEG microstates. NeuroImage 211:116631
